# INTERDISCIPLINARY PAIN REHABILITATION FOR IMMIGRANTS WITH CHRONIC PAIN WHO NEED LANGUAGE INTERPRETATION

**DOI:** 10.2340/jrm.v56.13466

**Published:** 2024-02-26

**Authors:** Karin UHLIN, Elisabeth PERSSON, Sofie BÄÄRNHIELM, Kristan BORG, Monika LÖFGREN, Britt-Marie STÅLNACKE

**Affiliations:** 1 Karolinska Institutet, Department of Clinical Sciences, Division of Rehabilitation Medicine, Danderyd Hospital; 2 Danderyd University Hospital, Department of Rehabilitation Medicine, Stockholm; 3 Centre for Psychiatry Research, Department of Clinical Neuroscience, Karolinska Institutet & Stockholm Health Care Services, Region Stockholm, Transcultural Centre, Region Stockholm; 4 Department of Community Medicine and Rehabilitation, Rehabilitation Medicine, Umeå University, Umeå, Sweden

**Keywords:** interdisciplinary pain rehabilitation, chronic pain, immigrants, language interpreter

## Abstract

**Objective:**

To investigate outcomes in patients with chronic pain after participation in an interdisciplinary pain rehabilitation programme with language interpreters, and to investigate the outcomes in women and men separately.

**Design:**

Prospective multi-centre cohort study.

**Patients:**

Ninety-five patients in Sweden with chronic pain who have insufficient knowledge of the Swedish language.

**Methods:**

Duration and intensity of pain, anxiety and depression, health-related quality of life and fear of movement were evaluated before and after the programme. Patients were compared with a reference group comprising Swedish-speaking patients participating in an ordinary interdisciplinary pain rehabilitation programme.

**Results:**

Before the interdisciplinary pain rehabilitation programme with language interpreters, all variables except pain duration differed significantly to the detriment of the studied group. The studied group showed significant improvements after the interdisciplinary pain rehabilitation programme with language interpreters, with regards to pain intensity, depression and fear of movement. The reference group improved significantly for all variables.

The women in the studied group showed significant improvements for the same variables as the whole group, while the men in the studied group did not improve in any of the variables.

**Conclusion:**

This study indicates that patients with chronic pain, and especially women, who have insufficient knowledge of Swedish seem to benefit from participating in an interdisciplinary pain rehabilitation programme with language interpreters. The result may be of value for the further development of rehabilitation programmes with language interpreters.

LAY ABSTRACT

Many people live with chronic pain that frequently causes suffering, since it often affects mood and the ability to cope with daily life. For immigrants, chronic pain can be an even greater problem, because they often experience other difficulties to a greater extent than does the native population. People with chronic pain may be offered rehabilitation. A method called interdisciplinary pain rehabilitation, comprising practical and theoretical interventions led by different professions, has helped many patients. However, to date, immigrants in Sweden with no knowledge of the Swedish language have been excluded from interdisciplinary pain rehabilitation. This study evaluates 2 interdisciplinary pain rehabilitation programmes in Sweden that included language interpreters, enabling immigrants with insufficient knowledge of the Swedish language to participate. The results indicate that interdisciplinary pain rehabilitation with language interpreters is feasible. It is hoped that more interdisciplinary pain rehabilitation programmes with language interpreters will be offered in future.

Chronic pain is a major health problem. Approximately 20% of the European population has moderate to severe pain conditions (1), and it has been shown that chronic pain is influenced by, and interacts with, physical, psychological, social and contextual factors (2).

People with chronic pain have described several problems caused by the pain, such as psychological distress (3) and limitations in performing everyday activities (4). In addition to impaired function and limitations in activity for the individual, these problems often lead to large socioeconomic costs, mainly due to the affected individuals’ limited ability to participate in working life (5).

A higher percentage of women than men experience chronic pain, with higher pain sensitivity and higher levels of distress connected to pain being found for women compared with men (6). Depression, which is reported to be approximately twice as common for individuals with pain compared with those without, is also more common among women than among men (7).

The complexity of chronic pain means that patients often need combined treatment based on a biopsychosocial approach. Several systematic reviews have revealed that interdisciplinary pain rehabilitation (IPR) programmes are more effective in reducing pain and restoring functions compared with single/unimodal interventions (8, 9). Most of the specialist pain clinics in Sweden that offer IPR programmes report data to the Swedish Quality Registry for Pain Rehabilitation (SQRP (https://www.ucr.uu.se).

The increase in immigration to Western countries poses a challenge to healthcare and rehabilitation (10). Migrants, particularly from non-western countries, are reported to have higher levels of pain intensity and more frequent musculoskeletal conditions (11) and an increased risk of mental health disorders (12) compared with native populations.

Swedish legislation states that the need for healthcare is the strongest principle of priority when selection for treatment is required (*Act of Health and Health Care, 1982*); thus, emphasizing the right to healthcare independent of sex, age, ethnicity and socioeconomic status. To overcome the language barrier patients in Sweden have the right to an interpreter, free of charge, at healthcare visits. All interpreters used in healthcare are legally bound by confidentiality and must translate everything that is communicated in the first person (13).

In Sweden, approximately 20% of patients with chronic pain referred to specialized pain rehabilitation centres are born abroad. Although there is evidence for IPR, foreign-born immigrants who do not speak Swedish are commonly not selected for participation in IPR (14). Hence, there is a lack of knowledge and studies on whether IPR can be used for patients who do not have sufficient knowledge of Swedish.

Therefore, 2 specialized pain rehabilitation centres in Sweden started to offer IPR programmes with language interpreters (IPR-LI) for patients with chronic pain who have inadequate skills in the Swedish language.

The primary aim of this study is to compare demographic data and baseline pain characteristics among the patients taking part in IPR-LI with a reference group of patients participating in an ordinary IPR programme (IPR-ORD) and to investigate the outcomes in both groups immediately after the programme. The secondary aim is to compare baseline scores for women and men separately with that of the reference group, and to investigate the outcomes separately for women and men.

## METHODS

This prospective multi-centre cohort study collected data from 2 university rehabilitation clinics in Sweden, the Department of Rehabilitation Medicine, Danderyd University Hospital, and the Department of Rehabilitation Medicine, Lund University Hospital, during 2013–18, through patient-completed questionnaires. Each patient who participated in the IPR-LI programme completed the questionnaires before and directly after taking part in the rehabilitation programme, with a selection of instruments from the SQRP including demographics, pain duration, location of pain, intensity of pain, anxiety and depression, health-related quality of life and fear of movement.

The questionnaires were sent home to the patients before they came to the clinic. If the questionnaires were not fully completed, the healthcare professionals helped the patient together with an interpreter. When the IPR-LI programme was finished, the patients completed the questionnaires with assistance from the healthcare professionals and interpreters.

### Questionnaires

SQRP questionnaires in Swedish were used to gather data on background information, pain characteristics, psychological factors and quality of life.

### Sociodemographic data

The following sociodemographic data were collected:

- age (years);
- sex (women, men);
- country of birth (Sweden, other Nordic country, European, non-European country);
- highest completed level of education (primary school, secondary school, higher education).

### Instruments

*Instruments for measuring pain*. Pain intensity over the previous week was measured using an 11-point numerical rating scale (NRS), with 0 representing “no pain” and 10 “worst pain imaginable”. The NRS constitutes a valid and sensitive measure (15).

The number of pain sites was registered using 36 predefined anatomical areas. The patient reported a number of sites with pain on the left side of the body (*n* = 18) and on the right side of the body (*n* = 18). These pain sites were: (1) head/face, (2) neck, (3) shoulder, (4) upper arm, (5) elbow, (6) forearm, (7) hand, (8) anterior aspect of chest, (9) lateral aspect of chest, (10) belly, (11) sexual organs, (12) upper back, (13) lower back, (14) hip/gluteal area, (15) thigh, (16) knee, (17) lower leg, and (18) foot..

Duration of pain was reported as the number of days since the pain started. The number of days was then recalculated to years, and this data are presented in Table I.

**Table I:** Demographics for interdisciplinary pain rehabilitation programme with language interpreters (IPR-LI) and ordinary interdisciplinary pain rehabilitation programme (IPR-ORD)

	IPR-LI *n* = 95	IPR-ORD *n* = 240
Sex, *n* (%)		
Female	74 (77.9)	196 (81.7)
Male	21 (22.1)	44 (18.3)
Age, years, mean (SD)	46.3 (8.9)	40.3 (11.3)
Median (min–max)	47.0 (24–64)	40.0 (18–65)
Education (*n* (%))		
Primary school only	23 (24.2)	21 (8.8)
Secondary school or vocational training	27 (28.4)	88 (36.7)
University	5 (5.3)	77 (32.1)
Other	8 (8.4)	21 (8.8)
Missing information	32 (33.7)	33 (13.8)
Country of origin, *n* (%)		
Born in Sweden	–	
Born in Nordic country	–	
Born in other European country	16 (16.8)	
Born outside of Europe	78 (82.1)	
Missing information	1 (1.1)	
Years in Sweden, mean (SD)	13.9 (8.2)	
Median (min–max)	11.0 (3–41)	
Missing information	7 (7.4)	

SD: standard deviation.

The number of pain sites and duration of pain were only reported before taking part in the IPR-LI.

*Hospital Anxiety and Depression Scale*. HADS is a measurement of anxiety (HADS-A) and depression (HADS-D) (16). The instrument consists of 7 items for anxiety and 7 items for depression. Each item can be rated from 0 to 3, whereby the respondents indicate how much it applied to them during the last week. The total scale, for each subscale of anxiety or depression, ranges between 0 and 21, with a higher score indicating a worse condition. A high score indicates the need for a clinical assessment for anxiety/depression. The obtained scores can be divided into 3 categories, where a score of 7 or lower indicates no anxiety/depression, a score of 8–10 indicates a mild disorder, and a score of 11 or higher is the cut-off for a possible clinically significant disorder (17).

*European Quality of Life 5 dimensions*. EQ-5D measures health-related quality of life (18). The instrument consists of 2 parts: 1 part measures health status in 5 dimensions (5D), mobility, self-care, usual activities, pain and discomfort, and anxiety and depression; the other part allows respondents to evaluate their overall health status using a 100-point scale (EQ-VAS), a vertical scale where the endpoint 100 is labelled “Best imaginable health state” and the endpoint 0 is labelled “Worst imaginable health state”. To be able to evaluate treatment results and make comparisons, the EQ-5D answers can be summed up in an index by weighting the scores, the EQ5D-Index (19).

*Tampa Scale of Kinesiophobia*. TSK measures perceived fear of (re)injury and movement on a 4-point Likert scale (1–4) with a total score between 17 and 68 (20). Higher scores indicate higher fear of movement and (re)injury. Satisfactory psychometric properties are found for both the original and the translated Swedish version (21).

### Interdisciplinary pain rehabilitation programme with language interpreters

Inclusion criteria for the IPR-LI-programme were age 18 years or over, disabling chronic pain, no further medical investigation needed, being a Swedish citizen or having a residence permit, and a need for a language interpreter due to insufficient knowledge of the Swedish language.

Exclusion criteria for the IPR-LI-programme were severe psychiatric disease, known drug abuse, and economic or social factors that were assessed as not being compatible with participation in the rehabilitation programme.

The patients were usually referred from primary care, but also from occupational healthcare and various specialist clinics. The IPR-LI programme was carried out by an interdisciplinary team that included a physician, an occupational therapist, a physiotherapist, a psychologist, and a social worker. One of the IPR-LI teams also included a nurse. The healthcare professionals were mostly native Swedish women. The programme consisted of interventions with a frequency of 2–3 days/week for up to 8 weeks with up to 8 patients and interpreters. The programme included pain physiology, pain management, psychoeducation and practical groups with physical training and occupation-based therapy and was based on principles from cognitive behavioural therapy (CBT) and acceptance and commitment therapy (ACT) (9). Individual visits were made, with a focus on formulating an individual rehabilitation plan with the setting of goals in the areas of activity, participation and coping with pain. Weekly team conferences were performed to monitor the progress of each patient.

Language interpreters were always present during the theoretical interventions and when necessary during practical interventions. The interpreters were scheduled and booked by the team secretary, thus rendering extra focus and time on administrative issues, especially since many nationalities and languages were represented among the patients.

The language interpreters were recruited in the normal manner, implying that different interpreters with differing qualifications were used in the programmes. To ensure the quality of interpretation the rules for interpreted mediated communication were read out loud to the interpreters and the patients when a new group started, the interpreters were trained in how rehabilitation methodology works, and a list of frequently used terms was sent in advance to the interpreter services.

If the interpretation did not seem to work during the conversation, the healthcare professionals tried to clarify themselves and also gave feedback to the interpreter after the conversation. The healthcare professionals adapted the material used in the ordinary programmes by simplifying and abbreviating both written and spoken texts and by adding pictures, to further optimize the opportunity for the patients to understand the content of the programme. The experiences of the healthcare professionals who implemented those programmes have been presented in an earlier study (22).

### Comparison with a reference group

Participants’ answers to the SQRP questionnaires were compared with those of a reference group from SQRP that comprised 240 patients participating in an ordinary IPR programme (IPR-ORD) at the Department of Rehabilitation Medicine, Danderyd University Hospital, Stockholm, during 2015–18.

### Ordinary interdisciplinary pain rehabilitation programme

The inclusion criteria for the IPR-ORD programme were the same as the inclusion criteria for the IPR-LI programme, except for criterion regarding the need for a language interpreter due to insufficient knowledge of the Swedish language.

The exclusion criteria for the IPR-ORD programme were the same as the exclusion criteria for the IPR-LI programme.

The description of the referring units, composition of the team, logistics and contents of the programme for the IPR-LI programme is also applicable to the IPR-ORD programme, except for the systematic use of language interpreters and the adaptation of the material used in the IPR-LI programme.

### Statistical analysis

Statistical analysis was performed with Statistic Package for Social Sciences software (SPSS), version 25. Data were reported both with means with standard deviations and medians with interquartile range. Descriptive analysis was used for the description of the population.

Differences between groups before IPR-LI and IPR-ORD were tested with Mann–Whitney *U* test. For analyses of the studied group and the reference group over time (before and after IPR-LI/IPR-ORD), Wilcoxon signed-ranks test was used. The statistical significance level was set as *p* < 0.05. The effect size was calculated using the effect size calculator for non-parametric tests (23), where eta-squared was calculated using the value z from Wilcoxon signed test. The interpretation is as follows: values around 0.01 are interpreted as small, values around 0.06 are interpreted as medium, and values over 0.14 are interpreted as large (24).

### Sample size

To test the hypothesis that the quality of life measured by the EQ5D-Index increased significantly from the start of treatment to after rehabilitation, 100 patients are required. This is based on a strength of 80%, a level of significance of 5% (2-sided), a standard deviation of the difference between the 2 times is 0.3 and an increase of a mean of 0.1, which corresponds to a previously measured, clinically significant change. With an estimated loss of 25%, 100 patients should be included.

### Ethics

The study was approved by the regional ethics review board Stockholm (ref: 2016/858-31 and completion 2016/2059-32 and 2020/06982).

## RESULTS

### Comparison of patients before interdisciplinary pain rehabilitation programme with language interpreters and patients before ordinary interdisciplinary pain rehabilitation programme

The demographics for the patients in IPR-LI, respectively IPR-ORD, are shown in Table I. There are significant differences between the groups for age (*p* < 0.001) and education (divided into 2 groups: primary school and secondary school plus university) (*p* < 0.001). Age is higher for the patients in IPR-LI. The proportion of patients with primary school as the highest education is higher in the IPR-LI, and the proportion of patients with secondary school or university as the highest education is lower in the IPR-LI (Table I).

The patients participating in IPR-LI had had pain for a long period, had a high number of pain sites, and rated high on pain intensity (NRS). The scores on HADS-A and HADS-D indicated possible clinically significant anxiety and depression disorders. The scores on EQ5D-Index indicated low health-related quality of life, and the scores on TSK indicated a high level of fear of movement/(re)injury (Table II).

**Table II:** Patient-reported outcome measures before interdisciplinary pain rehabilitation (IPR) with a language interpreter (IPR-LI) compared with a reference group (IPR-ORD)

	IPR-LI (*n* = 95) Before	Valid, *n*	IPR-ORD (*n* = 240) Before	Valid, *n*	*p*-value
Years with chronic pain, mean (SD)	7.6 (7.6)	87	8.9 (9.2)	227	0.771
Median (IQR)	4.5 (7.9)		5.5 (12.5)		
Number of pain sites, mean (SD)	20.4 (9.0)	95	16.4 (8.1)	240	< 0.001
Median (IQR)	21.0 (14.0)		16.0 (13.0)		
Pain intensity (last week) NRS, mean (SD)	8.4 (1.4)	94	6.7 (1.9)	228	< 0.001
Median (IQR)	8.5 (3.0)		7.0 (2.0)		
HADS-A, mean (SD)	13.2 (4.6)	91	10.2 (4.6)	237	< 0.001
Median (IQR)	14.0 (7.0)		10.0 (7.0)		
HADS-D, mean (SD)	11.4 (4.6)	91	9.6 (4.5)	238	0.002
Median (IQR)	12.0 (7.0)		9.0 (6.0)		
EQ5D-Index, mean (SD)	0.06 (0.26)	86	0.23 (0.30)	233	< 0.001
Median (IQR)	−0.02 (0.22)		0.12 (0.59)		
TSK, mean (SD)	50.8 (8.1)	78	40.1 (8.1)	231	< 0.001
Median (IQR)	51.0 (11.0)		39.0 (10.0)		

NRS: numerical rating scale for pain intensity; HADS-A and HADS-D: Hospital Anxiety and Depression Scale -Anxiety and -Depression; EQ5D-Index: European Quality of Life 5 dimensions index; TSK: Tampa scale of Kinesiophobia.

A comparison of patients in the IPR-LI and patients in the IPR-ORD before the programmes showed significant differences, to the detriment of the patients in the IPR-LI for pain intensity (NRS), number of pain sites, HADS-A, HADS-D, EQ5D-Index and TSK. No significant difference was seen between the groups for pain duration (Table II).

### Results for patients after interdisciplinary pain rehabilitation programme with language interpreters compared with results for patients after ordinary interdisciplinary pain rehabilitation programme

Pain intensity (NRS), depression (HADS-D) and fear of movement/(re)injury (TSK) decreased significantly for the patients participating in IPR-LI after the rehabilitation programme compared with before the programme. The effect sizes were medium for NRS and TSK, and small for HADS-D (Table III).

**Table III:** Comparison before and after interdisciplinary pain rehabilitation with a language interpreter (IPR-LI) and comparison before and after ordinary interdisciplinary pain rehabilitation (IPR-ORD)

	IPR-LI (*n* = 95) Before	IPR-LI After	Valid, *n*	IPR-LI after vs before *p*-value ES	IPR-ORD (*n* = 240) Before	IPR-ORD After	Valid	IPR-ORD after vs before *p*-value ES
Pain intensity (last week) NRS, mean (SD)	8.4 (1.4)	7.7 (1.7)	79	< 0.001	6.7 (1.9)	5.9 (1.9)	185	< 0.001
Median (IQR)	8.5 (3.0)	8.0 (2.0)		0.085	7.0 (2.0)	6.0 (2.0)		0.068
HADS-A, mean (SD)	13.2 (4.6)	12.8 (4.6)	88	0.328	10.2 (4.6)	8.4 (4.1)	203	< 0.001
Median (IQR)	14.0 (7.0)	13.0 (7.8)		0.006	10.0 (7.0)	8.0 (6.0)		0.095
HADS-D, mean (SD)	11.4 (4.6)	9.8 (4.8)	88	0.022	9.6 (4.5)	6.7 (3.9)	203	< 0.001
Median (IQR)	12.0 (7.0)	9.0 (6.0)		0.029	9.0 (6.0)	7.0 (5.0)		0.183
EQ5D-Index, mean (SD)	0.06 (0.26)	0.12 (0.33)	89	0.244	0.23 (0.30)	0.38 (0.33)	200	< 0.001
Median (IQR)	´−0.02 (0.22)	´−0.02 (0.63)		0.008	0.12 (0.59)	0.55 (0.60)		0.063
TSK, mean (SD)	50.8 (8.1)	46.6 (9.7)	75	< 0.001	40.1 (8.1)	33.9 (7.3)	199	< 0.001
Median (IQR)	51.0 (11.0)	48.0 (16.0)		0.098	39.0 (10.0)	33.0 (11.0)		0.245

NRS: numerical rating scale for pain intensity; HADS-A and HADS-D: Hospital Anxiety and Depression Scale -Anxiety and -Depression; EQ5D-Index: European Quality of Life 5 dimensions index; TSK: Tampa Scale of Kinesiophobia; ES: effect size; SD: standard deviation; IQR: interquartile range.

All variables decreased significantly in the reference group (IPR-ORD) after the rehabilitation programme compared with before the programme. The effect sizes were large for HADS-D and TSK, and medium for NRS, HADS-A and EQ5D-Index (Table III).

### Women and men before interdisciplinary pain rehabilitation programme with language interpreters compared with women and men before ordinary interdisciplinary pain rehabilitation programme

Comparisons between women in the IPR-LI and women in the IPR-ORD before the programmes showed significant differences, to the detriment of the women in the IPR-LI for pain intensity (NRS), number of pain sites, HADS-A, HADS-D, EQ5D-Index and TSK. No significant difference was seen between the groups for years with pain (Table IV).

**Table IV:** Patient-reported outcome measures before interdisciplinary pain rehabilitation programme with language interpreters (IPR-LI) in women (IPR-LIW) and men (IPR-LIM) compared with women (IPR-ORDW) and men (IPR-ORDM) respectively in a reference group

	IPR-LIW (*n* = 74) Before	Valid *n*	IPR-ORDW (*n* = 196) Before	Valid *n*	IPR-LIW vs IPR-ORDW Before *p*-value	IPR-LIM (*n* = 21) Before	Valid *n*	IPR-ORDM (n = 44) Before	Valid *n*	IPR-LIM vs IPR-ORDM Before *p*-value	IPR-LIW vs IPR-LIM Before *p*-value
Years with chronic pain, mean (SD)	7.6 (7.7)	68	9.4 (9.5)	184	0.482	7.7 (7.6)	19	6.8 (7.4)	43	0.397	0.781
Median (IQR)	4.3 (7.8)		6.3 (12.6)			6.0 (9.9)		4.0 (8.4)			
Number of pain sites, mean (SD)	21.7 (8.7)	74	17.3 (8.2)	196	< 0.001	15.6 (8.6)	21	12.5 (6.3)	44	0.156	0.006
Median (IQR)	22.0 (12.3)		16.5 (12.8)			16.0 (12.5)		12.5 (11.8)			
Pain intensity (last week) NRS, mean (SD)	8.5 (1.3)	74	6.7 (1.8)	187	< 0.001	8.1 (1.7)	20	6.8 (2.0)	41	0.021	0.380
Median (IQR)	9.0 (2.0)		7.0 (2.0)			8.0 (3.0)		7.0 (2.0)			
HADS-A, mean (SD)	13.3 (4.8)	71	10.0 (4.5)	194	< 0.001	12.8 (3.9)	20	11.0 (4.7)	43	0.143	0.462
Median (IQR)	14.0 (7.0)		10.0 (8.0)			12.0 (5.9)		11.0 (7.0)			
HADS-D, mean (SD)	11.2 (4.5)	71	9.5 (4.4)	194	0.004	12.0 (4.8)	20	10.3 (5.0)	44	0.179	0.501
Median (IQR)	12.0 (6.0)		9.0 (5.5)			11.5 (6.9)		10.5 (7.0)			
EQ5D-Index, mean (SD)	0.06 (0.25)	68	0.24 (0.29)	190	< 0.001	0.03 (0.30)	18	0.19 (0.32)	43	0.026	0.452
Median (IQR)	´-0.02 (0.24)		0.16 (0.59)			´-0.07 (0.24)		0.09 (0.69)			
TSK, mean (SD)	50.6 (8.4)	60	39.6 (8.0)	187	< 0.001	51.2 (6.8)	18	42.3 (8.6)	44	< 0.001	0.859
Median (IQR)	50.0 (11.0)		39.0 (10.5)			52.5 (10.3)		42.0 (12.4)			

NRS: numerical rating scale for pain intensity; HADS-A and HADS-D: Hospital Anxiety and Depression Scale -Anxiety and -Depression; EQ5D-Index: European Quality of Life 5 dimensions index; TSK: Tampa Scale of Kinesiophobia; IPR-ORD: Ordinary interdisciplinary pain rehabilitation programme.

Comparisons between men in the IPR-LI and men in the IPR-ORD before the programmes showed significant differences to the detriment of the men in the IPR-LI for pain intensity (NRS), EQ5D-Index and TSK. No significant differences were seen between the groups for years with pain, number of pain sites, HADS-A and HADS-D (Table IV).

A comparison of women and men in the IPR-LI before the programme showed a significant difference, to the detriment of the women for number of pain sites. No significant differences were seen between the groups for years with pain, pain intensity (NRS), HADS-A, HADS-D, EQ5D-Index and TSK (Table IV).

A comparison of women and men in the IPR-ORD before the programme showed a significant difference to the detriment of the women for number of pain sites (*p* < 0.001) and TSK (*p* = 0.044). No significant differences were seen between the groups for years with pain, pain intensity (NRS), HADS-A, HADS-D and EQ5D-Index.

### Women and men after interdisciplinary pain rehabilitation programme with language interpreters compared with women and men after ordinary interdisciplinary pain rehabilitation programme

Pain intensity (NRS), depression (HADS-D) and fear of movement/(re)injury (TSK) decreased significantly for the women participating in IPR-LI after the rehabilitation programme compared with before the programme. The effect sizes were medium for NRS and TSK, and small for HADS-D (Table V).

**Table V:** Change in patient-reported outcome measures after interdisciplinary pain rehabilitation programme with language interpreters (IPR-LI) for women (IPR-LIW) and after Ordinary interdisciplinary pain rehabilitation programme (IPR-ORD) for women (IPR-ORDW)

	IPR-LIW (*n* = 74) Before	IPR-LIW After	Valid *n*	IPR-LIW after vs before *p*-value ES	IPR-ORDW (*n* = 196) Before	IPR-ORDW After	Valid *n*	IPR-ORDW after vs before *p*-value ES
Pain intensity (last week) NRS, mean (SD)	8.5 (1.3)	7.7 (1.6)	62	< 0.001	6.7 (1.8)	6.0 (1.8)	156	< 0.001
Median (IQR)	9.0 (2.0)	8.0 (2.0)		0.104	7.0 (2.0)	6.0 (2.0)		0.048
HADS-A, mean (SD)	13.3 (4.8)	12.5 (4.7)	69	0.129	10.0 (4.5)	8.5 (4.0)	171	< 0.001
Median (IQR)	14.0 (7.0)	12.8 (7.0)		0.017	10.0 (8.0)	8.0 (6.0)		0.077
HADS-D, mean (SD)	11.2 (4.5)	9.5 (4.7)	69	0.020	9.5 (4.4)	6.8 (3.8)	171	< 0.001
Median (IQR)	12.0 (6.0)	9.0 (5.0)		0.039	9.0 (5.5)	7.0 (5.0)		0.18
EQ5D-Index, mean (SD)	0.06 (0.25)	0.12 (0.31)	71	0.312	0.24 (0.29)	0.38 (0.33)	169	< 0.001
Median (IQR)	´−0.02 (0.24)	´−0.02 (0.27)		0.008	0.16 (0.59)	0.52 (0.60)		0.053
TSK, mean (SD)	50.6 (8.4)	46.1 (9.2)	58	< 0.001	39.6 (8.0)	33.7 (7.4)	167	< 0.001
Median (IQR)	50.0 (11.0)	47.5 (14.0)		0.106	39.0 (10.5)	33.0 (11.0)		0.244

NRS: numerical rating scale for pain intensity; HADS-A and HADS-D: Hospital Anxiety and Depression Scale -Anxiety and -Depression; EQ5D-Index: European Quality of Life 5 dimensions index; TSK: Tampa Scale of Kinesiophobia; ES: effect size.

All variables decreased significantly for the women participating in the reference group (IPR-ORD) after the rehabilitation programme compared with before the programme. The effect sizes were large for HADS-D and TSK, medium for HADS-A, and small for NRS and EQ5D-Index (Table V).

None of the variables decreased significantly for the men participating in IPR-LI after the rehabilitation programme compared with before the programme (Table VI).

**Table VI:** Change in patient-reported outcome measures after interdisciplinary pain rehabilitation programme with language interpreters (IPR-LI) for men (IPR-LIM) and after Ordinary interdisciplinary pain rehabilitation programme (IPR-ORD) for men (IPR-ORDM)

	IPR-LIM (*n* = 21) Before	IPR-LIM After	Valid *n*	IPR-LIM after vs before *p*-value ES	IPR-ORDM (*n* = 44) Before	IPR-ORDM After	Valid *n*	IPR-ORDM after vs before *p*-value ES
Pain intensity (last week) NRS, mean (SD)	8.1 (1.7)	7.4 (2.3)	17	0.238	6.8 (2.0)	5.3 (2.0)	29	< 0.001
Median (IQR)	8.0 (3.0)	8.0 (3.5)		0.041	7.0 (2.0)	5.0 (2.5)		0.234
HADS-A, mean (SD)	12.8 (3.9)	14.1 (4.5)	19	0.458	11.0 (4.7)	8.1 (4.5)	32	< 0.001
Median (IQR)	12.0 (5.9)	15.0 (8.0)		0.014	11.0 (7.0)	7.0 (7.3)		0.217
HADS-D, mean (SD)	12.0 (4.8)	10.8 (5.3)	19	0.652	10.3 (5.0)	6.4 (4.4)	32	< 0.001
Median (IQR)	11.5 (6.9)	11.0 (9.0)		0.005	10.5 (7.0)	6.5 (7.5)		0.196
EQ5D-Index, mean (SD)	0.03 (0.30)	0.11 (0.39)	18	0.538	0.19 (0.32)	0.41 (0.34)	31	0.003
Median (IQR)	´-0.07 (0.24)	0.05 (0.80)		0.011	0.09 (0.69)	0.62 (0.66)		0.141
TSK, mean (SD)	51.2 (6.8)	48.3 (11.4)	17	0.154	42.3 (8.6)	35.0 (6.9)	32	< 0.001
Median (IQR)	52.5 (10.3)	52.0 (20.0)		0.06	42.0 (12.4)	34.0 (8.8)		0.248

NRS: numerical rating scale for pain intensity; HADS-A and HADS-D: Hospital Anxiety and Depression Scale -Anxiety and -Depression; EQ5D-Index: European Quality of Life 5 dimensions index; TSK: Tampa Scale of Kinesiophobia; ES: effect size.

All variables decreased significantly for the men in the reference group (IPR-ORD) after the rehabilitation programme compared with before the programme. The effect sizes were large for all the variables (Table VI).

## DISCUSSION

The results of this study show that patients participating in IPR-LI scored significantly less favourably in all baseline characteristics, except for pain duration, compared with the reference group. The findings after rehabilitation showed that patients participating in IPR-LI improved significantly in pain intensity, depression and fear of movement, while the reference group improved in all variables. A subgroup analysis of women and men in the IPR-LI group showed that the improvement at the group level was due to the women’s improvement.

The fact that the IPR-LI group showed a significantly worse health situation before the programme compared with the reference group is in line with other studies of patients participating in interdisciplinary pain rehabilitation programmes (25, 26) and studies of immigrant patients with pain (27). In a Swedish population-based study (27), a poor health-related situation for immigrant patients was described, including an increased risk for immigrants of developing different sorts of pain, mediated by their state of mood. In a study based on the SQRP registry, patients who were born outside Europe, of both sexes and different educational levels, were found to be the group with the poorest situation before the programme, based on pain intensity, depression, anxiety and health-related quality of life (25). Also, when the different patient groups are European, the non-native patients have been shown to have less favourable health (26). In this Swiss study by Benz et al. (26), immigrant patients (Italian-speaking) showed worse self-reported health compared with native patients (German-speaking) before the programme.

The IPR-LI programme can be argued to be costly, due to interpreters and extra administration. However, the current study indicates that IPR might be a workable rehabilitation method for immigrants with pain when a language interpreter is used; the patients who showed a difficult health situation before the programme improved significantly with regard to pain intensity, depression and fear of movement. Hypothetically, one could argue that these improvements ought to be reflected in an improvement in EQ5D-Index. However, EQ5D-Index might not be sensitive enough to capture the relatively small changes (compared with the reference group) seen after the IPR-LI rehabilitation. The improvements in the IPR-LI group are in line with the study by Benz et al. (26), where both the native (German-speaking) and the immigrant (Italian-speaking) patients improved after rehabilitation, but the native patients improved the most. Language interpreters were not used in that study since the healthcare professionals involved in the programme were German-speaking and Italian-speaking, respectively.

Earlier research shows inconclusive patterns concerning an unfavourable baseline situation and rehabilitation outcome. In a study based on the SQRP registry (28), the subgroup with the poorest situation at baseline showed the largest improvements after the IPR programme. A different result was reported from the study based on the SQRP registry referred to above (25), where the patients with the best baseline measures showed the largest improvements after the programme. In contrast with the current study, all patients in those studies, including those labelled as “born outside Europe”, participated in programmes corresponding to IPR-ORD, and consequently must have had a sufficiently good knowledge of Swedish to take part.

It is clearly stated in all IPR programmes that the goal per se is improved coping with pain and not diminished pain. However, it is important to try to reduce pain intensity because of a demonstrated positive correlation with disability (29). Several studies of outcomes after IPR include pain intensity, and it has been found that changes in pain intensity, pain interference, psychological distress and vitality are positively correlated (30), and therefore it is possible to assume that the improvements in depression and fear of movement in the IPR-LI group contributed to decreased pain intensity in the group.

There could be a number of reasons why the IPR-LI group did not improve in all the measured variables. Having a migration background has been found to correlate significantly with a less favourable outcome after participation in rehabilitation (31). The low level of education for the studied group might be one explanation. Level of education and knowledge about the body and pain are positively correlated (32) and it is also possible that a low level of education might be associated with less functional coping strategies to handle the pain condition once affected (33). In addition, very high levels of psychological problems may make it too difficult to benefit from an IPR programme (34), which could apply to some of the patients in the IPR-LI group. Differences due to cultural background between patients and healthcare professionals in the IPR-LI, impacting important areas, such as views about pain and goalsetting (22), may also have played a role in the outcome for the patients.

A comparison of baseline values for women and men in the IPR-LI group revealed that only pain locations showed a significant difference, with women reporting more locations. This difference between the sexes is well-known and, in this study, the same difference was seen in the reference group. However, there might be differences between women and men in variables not investigated in this study. In a study that investigated differences between women and men when entering an IPR programme (35), it was found that, when experiencing the same pain severity, women reported significantly higher activity levels, pain acceptance and social support while men reported significantly higher kinesiophobia, mood disturbances and significantly lower activity levels.

The women in the IPR-LI group improved significantly in pain intensity, depression and fear of movement, in contrast to the men in the IPR-LI group, who did not improve in any of the measured variables. Earlier studies evaluating ordinary IPR programmes have also shown that women improve to a larger extent than men (25, 36).

The conflict for men between a traditional male role and the state of a chronic condition was investigated in a study by Flurey et al. (37) of men with rheumatoid arthritis (RA), where the research group in a multi-centre survey confirmed their findings from earlier studies. The study established that, for a large group of men, it was difficult to find a new transformed identity. Also, men and women were found to use different coping strategies, and, as support, the men preferred information rather than sharing and discussing with other patients, as well as focusing on medical and practical aspects rather than psychological aspects. In addition, the process of acculturation of migrants (38) may be different for men and women. In a study examining sex-related aspects in rehabilitation for chronic pain (39), healthcare professionals, especially male healthcare professionals, expressed that the IPR programmes offered seemed more suitable for women than for men. Those findings suggest that men may need rehabilitation methods other than the ones traditionally used, which could be part of the explanation as to why the men in the current study did not improve after the programme. There is a need to further examine such aspects for men with chronic pain, and to include men from migrant populations.

### Methodological considerations

To the best of our knowledge, the setup with interdisciplinary pain rehabilitation in a group with a language interpreter has not been investigated before in Sweden and there are few studies from other countries. The multi-centre approach made it possible to collect data from a larger group of patients and will enable the result to be applied in a wider context. The size of the group also made it possible to subgroup, which revealed sex-related differences.

The participants answers to the SQRP questionnaires were compared with a reference group of patients participating in an ordinary IPR programme, since the study was conducted in a clinical setting and it was not possible to use a matched control group.

There are problems when using questionnaires and instruments in Swedish with migrant patients in need of interpreters. This is due to linguistic translation challenges and problems with cultural validity when using instruments in cross-cultural situations (40). In addition, there are culturally differing perceptions of an estimation situation; for example, that communication about symptoms can be culturally coded expressions of psychopathology, distress and social discontent (41).

A healthcare professional and an interpreter were present to support the patients in understanding the questionnaires. Using forms in the patients’ primary languages was not an alternative, since there were validated translations for only a few of the forms into relatively few languages.

Language interpretation is complicated and there is a risk that the patient-clinician communication is not correctly translated or that linguistic nuances are missed, even with an interpreter, which is an extra challenge for the healthcare professionals (22), who have to be aware of the risk of misunderstandings and incorrect measures. However, to receive safe care and an effective rehabilitation process, it is important to use an interpreter when needed (42).

The response rate was low for some of the instruments, as indicated in the tables or text.

In conclusion, even though patients with chronic pain with inadequate knowledge of Swedish had more severe problems than patients participating in an ordinary IPR programme, the patients, and especially the women participating in IPR-LI seemed to benefit from the intervention. Since men did not improve at all after IPR-LI, they may need further support during the programme. These findings may be of value for the further development of rehabilitation programmes with language interpreters, but additional studies with longer follow-up times are needed.
